# Effort and control of pain: a dilemma for patients and providers

**DOI:** 10.1097/PR9.0000000000001468

**Published:** 2026-07-10

**Authors:** John A. Sturgeon, Matthew B. Jennings, Zina Trost

**Affiliations:** a Department of Anesthesiology, University of Michigan Medical School, Ann Arbor, MI, USA; b Thomas Roth Sleep Disorders and Research Center, Henry Ford Health, Detroit, MI, USA; c Department of Psychological and Brain Sciences, Texas A&M University, College Station, TX, USA

## Abstract

**Commentary on:** Habermann MB, Christian MB. Uncontrollability of experimental pain decreases pain intensity and relief pleasantness ratings in a between- group design. PAIN Rep 2026;11:e1448.

Habermann^14^ presents an interesting extension of learned helplessness paradigms into experimental pain research by examining a pain avoidance task—where pain was controllable in the control group but not in the experimental group—with a test phase in which behavioral efforts could indeed reduce pain. Learned helplessness is well-validated in the depression literature^1^ and has informed clinical models of chronic pain for decades^9,28,29^ but research on generalization of learned pain-related helplessness into new contexts is relatively limited.

The study design is based on solid theoretical inference. It tests an assumption that participants initially unable to avoid pain may adopt a more passive, low-effort approach that would carry into the subsequent phase, when pain *can* be avoided. This hypothesis appears partially supported, with indications of disengagement by experimental participants (ie, those without initial control) as reflected by attenuated ratings of both pain and relief relative to the control group. However, this study noted only modest differences between the control and experimental conditions during the test phase, suggesting that generalizations of learning to a new context was inconsistent or heterogenous. Such heterogeneity in behavioral responses is perhaps unsurprising when considered against the broader literature on pain-related learning and adaptation, which likewise reveals considerable variability across individual responses.^16,22,27^

The authors thoughtfully suggest their findings may have relevance for chronic pain. Indeed, is there a “correct” way to regulate effort in the face of uncontrollable pain? In the context of this commentary, “effort” denotes a meaningful expenditure of energy to adapt cognitive or behavioral responses to reduce pain or maintain or improve function. Examining how people learn from and regulate their responses to pain may help identify an answer.

Many with chronic pain struggle to control the intensity of pain,^31^ often adjusting behavioral efforts around the perceived threat of pain flares.^8^ In experimental settings, individuals with chronic low back pain may overestimate how much effort they exert in physical tasks^11^ and show a decreased willingness to expend effort when greater reward is paired with greater pain risk.^2,10^ Clinically, such behaviors are commonplace and often maladaptive as patients withdraw from important life activities,^2^ potentially reducing responsiveness to reward and reinforcing longer-term disability.^21^ Problems also arise with excessive behavioral effort. Some individuals push through pain, ignoring pain signals and suppressing pain-related distress to maintain function. Patterns of *overexertion* or *overactivity* develop in the face of persistent or elevating pain, leading to subsequent periods of incapacitation.^15^ While this response can preserve function in the short term, it can actually foster feelings of helplessness and sustain chronic pain over time.^15^

Approaches to regulating effort are similarly diverse across behavioral treatments for pain. Cognitive-behavioral therapy (CBT), the most ubiquitous and widely studied behavioral pain treatment, teaches patients to preemptively modulate behavioral effort in anticipation of pain. Its strategies aim to strike a balance between behavioral withdrawal and overexertion. Activity pacing, for example, encourages pursuit of important life activities without pushing to the point of substantially worsening symptoms. Yet these techniques are not universally effective. Although pacing has clear face validity, it can become maladaptive when pain signals push patients away from valued pursuits or foster patterns of avoidance, worsening pain and function over time.^3,19,23^ Pacing may also contradict other pain-relevant behavioral strategies. For instance, insomnia treatments discourage daytime rest so as to build sleep pressure throughout the day. As sleep disturbance is a robust predictor of long-term pain-related outcomes,^30^ clinicians may offer competing advice about when to rest, when to persist, and which symptom(s) *should* guide behavioral effort.

Acceptance and commitment therapy (ACT) emphasizes psychological flexibility as a core tenet of chronic pain treatment. ACT explores past efforts to control pain, particularly when these efforts were ineffective or unduly costly to overall quality of life. When these efforts keep people stuck or pull them away from valued life activities, ACT encourages a different response. Rather than trying to control pain and distress, patients practice reducing behavioral or experiential avoidance and learn to reduce reactivity to aversive states such as pain or negative emotions so as not to prolong or exacerbate them.^13^ Acceptance and commitment therapy is also conducive to behavioral techniques, such as cycling or activity pacing, when such techniques help someone behave in line with personal values.^32^ Recent efforts have also shown promise in combining CBT and ACT principles, pairing pacing with flexible coping and acceptance.^4^

Mindfulness-based approaches, such as mindfulness-based stress reduction or mindfulness-based cognitive therapy, frame pain somewhat differently. In these models, pain triggers reflexive (ie, automatic) responses that erode quality of life and worsen distress and dysfunction. Effort therefore takes a different form. While development of regular mindfulness practice is deliberate, meditative practices deemphasize effortful attempts to control unwanted sensory, cognitive, or emotional experiences while encouraging focused, nonstriving awareness of the present moment.^7,18^ Cognitive effort may still manifest during meditation in subtler ways, for example, shifting or reorienting attention to a chosen anchor.^18^

Notably, integration of both effortful and less control-oriented techniques for behavioral and cognitive effort has shown significant promise in pain treatment. Clinical hypnosis, which can be effective for both acute and chronic pain, reflects an interplay of effortful and more flexible cognitive responses.^25^ Although approaches vary, hypnotic induction for pain is often initiated in a purposeful, effortful way (through focused attention and relaxation). Subsequently, hypnotic suggestions are delivered in a more open and less directive process (eg, suggestions about changing pain, one's relationship to pain, bolstering of positive states such as self-confidence) that are adopted based on what resonates with the patient.^12,24^

Similarly, emerging psychotherapies for nociplastic pain such as Pain Reprocessing Therapy (PRT) and Emotional Awareness and Expression Therapy (EAET) co-opt several of the techniques described above. These therapies maintain a central goal of facing interoceptive signals, including pain and emotion, openly while reappraising them as safe and healthy rather than attempting to avoid or control them. In PRT, a key technique is somatic tracking, in which patients orient attention toward pain without attempting to control or eliminate it, while reminding themselves that pain is *not* dangerous and the body is safe. Over time, this practice is extended in a graded fashion to more painful or fear-provoking situations so that safety learning generalizes, akin to CBT approaches for panic disorder.^5^ Emotional Awareness and Expression Therapy adopts a similar approach but targets emotional experiences, particularly those that have been long suppressed or avoided. Patients first identify patterns of emotional suppression or avoidance that may have emerged within the context of chronic stress, interpersonal conflict, or trauma. Patients subsequently learn to drop these defenses and allow emotions to be experienced openly rather than suppressed as a means of reducing brain responses that are generating or maintaining pain. From there, treatment moves toward progressively fuller emotional disclosure through difficult techniques such as expressive writing, Gestalt empty chair exercises, and assertiveness training—interpersonal strategies aimed at resolving ongoing sources of interpersonal and emotional conflict. Accordingly, both PRT and EAET seek a balance between periods of less-effortful awareness and openness (as in mindfulness meditation) and periods of more effortful exposure to avoided stimuli (as in exposure therapies), ideally through the lens of pursuing personally valued activities (as in ACT).

It should be acknowledged that conceptualizations of effort overlap across therapeutic approaches. ACT regularly incorporates behavioral techniques from CBT, while also conceptualizing mindfulness as a core tenet.^20^ Mindfulness-based therapies, which are largely nondirective, can improve pain and emotional distress but may require more structured behavioral or cognitive principles to meaningfully change behavioral patterns tied to future functional improvement.^20,26^ Protocols integrating hypnosis with CBT principles have similarly emerged as a highly promising chronic pain treatment.^17^

To be clear, this commentary is not intended to rank the relative value of one therapeutic approach over another, but to contrast how each addresses effort. Chronic pain is frustrating and often uncontrollable, leaving patients with difficult questions about how much effort to spend and when to spend it. Too little effort may pull patients away from the activities and people they value. Too much effort, or effort applied too rigidly without clear direction, may worsen pain and life in general. Furthermore, those with chronic pain may experience stigma or invalidation from others who interpret efforts to modify activities, set realistic expectations, or pace oneself as laziness, unreliability, or avoidance.^6^ The wide range of ways in which behavioral pain treatments address effort reflects the inherent difficulty of chronic pain: what can patients do when effort fails to yield relief? What should they do when “trying harder” only makes matters worse? Is there a “correct” way to respond to uncontrollable pain? Clinical and empirical models must keep developing in ways that account for effort, its antecedents (social and goal context, medical and psychological contributors), and its downstream effects (postexertional flares, affective distress, social responses from others).

Habermann's study ends where our treatments so often begin, with a question of where to spend effort to reduce pain and its impact on life. Individuals facing periods of uncontrollable pain do not respond uniformly to this loss of control, and real-world treatments must be responsive to the wide range of adaptive effort underlying these responses.

## Disclosures

The authors have no conflict of interest to declare.
